# American Veterinary Journal

**Published:** 1859-05

**Authors:** 


AMERICAN VETERINARY JOURNAL.—One year ago we took occasion to speak of this journal and to commend it to the notice of our readers, expressing our conviction that a periodical of this kind, edited by a competent gentleman, was much needed. We have now to record its suspension, the reason for which will be readily seen from the following pointed circular of Dr. Dadd. The subscription was but one dollar a year:—

"I take the liberty to inform you that the publication of the 'AMERICAN VETERINARY JOURNAL' is suspended with the March issue. The only explanation I have to offer is, that in consequence of remissness on the part of subscribers for the past two years, my pocket-book is now the seat of a very severe attack of dyspepsia, which threatens to confine me and my family to a diet of shorts."

